# Spatial Information in the Emergence of Life

**DOI:** 10.3389/fgene.2021.672780

**Published:** 2021-09-09

**Authors:** Hugo I. Cruz-Rosas, Pedro Miramontes

**Affiliations:** Facultad de Ciencias, Universidad Nacional Autónoma de México, Ciudad Universitaria, Mexico City, Mexico

**Keywords:** self-propagating conformation, adaptive prebiotic systems, biological information, origins of life, origins of biochirality

## Abstract

Information in living systems is part of a complex relationship between the internal organization and functionality of life. In a cell, both genetic-coding sequences and molecular-shape recognition are sources of biological information. For folded polymers, its spatial arrangement contains general references about conditions that shaped them, as imprints, defining the *data* for spatial (conformational) information. Considering the origin of life problem, prebiotic dynamics of matching and transfer of molecular shapes may emerge as a flow of information in prebiotic assemblages. The property of carrying information in molecular conformations is only displayed at this system organization level. Accordingly, spatial information is a resource for active system responses toward milieu disturbances. Propagation of resilient conformations could be the substrate for structural maintenance through dynamical molecular scaffolding. The above is a basis for adaptive behavior in potentially biogenic systems. Starting from non-structured populations of carrying-information polymers, in the present contribution, we advance toward an entire theoretical framework considering the active role of these polymers to support the emergence of adaptive response in systems that manage conformational information flow. We discuss this scenario as a previous step for the arising of sequential information carried out by genetic polymers.

## Introduction

Biological adaptation is an evolutionary response that depends on a flow of information that links the organization with functions in living systems, according to the changing environmental context. In this sense, biological adaptation works like the construction of relationships between life and surroundings (Lewontin, 1981). Through molecular complexes, organisms can sense the external conditions to trigger a function supported by the system architecture. This correspondence between conditions and system behavior assumes the existence of information. Currently, there is no unique definition of this concept. However, we have identified five criteria to consider the presence of information in a dynamic: (1) There must exist the self-choice of the following system state (self-controlled development); (2) there is distinguishability between elements, so uncertainty is reduced; (3) some system elements have a meaning in the context of the dynamic, so they represent (correlate with) other elements or processes; (4) the interpretation of these elements is possible in the context of the dynamic; and (5) the set of data (references that carry meaning) in these elements exerts influence on the formation of patterns or processes in the system, so these data can be stored and transmitted (Cruz-Rosas et al., 2020).

Considering the cell functionality, there are two biological information sources: The cryptic, supplied by sequences in genetic polymers; and spatial information, explicitly transferred between molecular geometries like molds (Cruz-Rosas et al., 2020). Explicit spatial information is directly available from a source in the system dynamic without using a transducer to decode it. Folding on polymers has geometrical data that correlate with the general environmental context that has converged to shape it. Three-dimensionally structured polymers are information carriers concerning milieu condition intervals. Regarding supramolecular recognition, spatial information is defined as one that originated from the molecular shape discrimination as different information-carrying entities by employing data (metric relationships) contained in its geometrical arrangements. Regarding folded polymers, the spatial information is *conformational* (Maury, 2015; Cruz-Rosas et al., 2020). The self-propagating capability and relative simplicity of this conformational information have been pointed out as supporting plausible biogenic dynamics (Ogayar and Sánchez-Pérez, 1998; Maury, 2015, 2018; Cruz-Rosas et al., 2017, 2020). Theoretically, the spreading of resilient conformations among prebiotic peptides allows the structural counteraction of milieu perturbations, defining a starting point for an active response toward surroundings by prebiotic systems (Cruz-Rosas et al., 2017; Kompanichenko, 2017a).

In a previous work (Cruz-Rosas et al., 2017), we have shown the pertinence for considering peptides as spatial-information carrying polymers. Besides, we also have theoretically shown the fundamental role of chiral asymmetry by making the flow of conformational information functional. In the present contribution, we expand our theoretical approach to the system organization level. The main outcome is the emergence of adaptive behavior by incorporating the chiral bias in the propagating conformations.

Consequently, in a systemic overview, we argue that the presence of a diversity of resilient conformations in peptides allows the buffering of diverse milieu conditions, supporting an adaptive behavior (not yet in a biological sense) in potentially biogenic systems. Resilient conformations are not easily destabilized, so their peptide populations in the milieu do not collapse under harsh conditions, working as a reservoir of structures for establishing interactions with surroundings at different environmental times when incorporated in the systems. Conformations on members of these reservoirs can self-propagate when the milieu retracts pressure over its spatial stability while inhibiting the spread of other resilient conformations. If this flow of conformational information is incorporated into system architecture, structural maintenance is a possible lasting-time response. Accordingly, we discuss spatial information’s role in the emergence of adaptive behavior as a previous step to developing functional coding sequences that lead to the emergence of life.

## Statements and Discussion

To propose a general theoretical framework for the emergence of biogenic systems, we expand our previous works for the origin of life addressed from the critical role of peptides with inner chiral bias to their response toward milieu disturbances (Cruz-Rosas et al., 2017). Also, the informational character in the molecular shape as a source of biological information is assumed (Cruz-Rosas et al., 2020). The current work evaluates our hypotheses at the system level of organization, so this study brings new insights about the implications of the conformational information supporting collective behaviors on molecular assemblages. We present below the arguments for the emerging dynamical scaffolding capable of encouraging adaptive behavior on prebiotic systems as a previous step for the emergence of sequential-genetic information. The statements below represent the theoretical ground for the necessary searching for evidence employing mathematical or *in silico* models, opening some new perspectives in researching the origin of life.

### Prebiotic Adaptive Behavior and Spatial Information

Adaptive behavior is a collective property that can result from a flow of conformational information. Molecular adaptive systems are structured through interdependent entities working as an integrated whole capable of responding toward internal or external changes. Adaptation allows systems to develop different interactions with concrete surrounding contexts and change their organization and properties toward the changing milieu (Nechansky, 2010). The system unit’s diversification and temporal continuity are the main consequences of this adaptive behavior (Lewontin, 1983). Accordingly, the relations between the surroundings and the system through concrete molecular constituents give rise to a mutual interplay. The resulting molecular structuring from these interactions (the adoptions of a molecular shape) makes possible a representation of the environmental conditions available for the system. Working at a supramolecular level, the shape-structuring on the system constituents represents a spatial information source for prebiotic dynamics (Cruz-Rosas et al., 2020). The emergence of potentially biogenic systems supported by this type of explicit information has been theoretically addressed (Ogayar and Sánchez-Pérez, 1998; Maury, 2015, 2018; Cruz-Rosas et al., 2017, 2020). An essential result in those works is that molecular geometries correlate with concrete milieu conditions because a set of parameters converged on their spatial structuring. However, the implications of the above at the system level of organization are not explored. Accordingly, an evaluation at this organizational level is needed because the explicit information in polymer conformations is only an active feature in the context of their interactions when considering an informational dynamic.

The systemic overview shows that the correlations between the spatial arrangement in the components and the environmental conditions are the basis for the system’s interplay with surroundings. Accordingly, an active response toward the environment is prepared to emerge with simple functions such as buffering external perturbations employing the structured components to absorb energy from disturbances. Therefore, when assembled, the systems alter their states with the same probability as the change induced by the milieu stimuli (Martín et al., 2009). Molecular components not establishing connections with other molecules may already absorb energy from surroundings, so they are potential entities to construct interactions with the milieu. Their capability to interact and correlate with environmental parameters makes them the elements to carry information in a system. Hence, molecular assemblies allow that components construct simple connections between them through supramolecular interactions. The spatial structuring of polymers capable of inducing a conformational change over other polymers and propagating a particular shape is the basis for establishing an information flow that creates cohesion as a unity. The spreading of such structuring in a spatial informational dynamic is critical for constructing adaptive behavior on a prebiotic system.

The theoretical approach of spatial information is not restricted to polymers. However, it is a general hypothesis to consider as a starting point for an information system that incorporates components that correlate with milieu conditions, carrying information about it. The structuring of molecular complexes driven by environmental conditions has been addressed in proposals such as those considering UV irradiation, which can induce the assemblage of nucleobases working as chromophores capable of absorbing and dissipating photon energy (Michaelian, 2011, 2017). Likewise, molecular structuring that gives way to system scaffolding components has been proposed to buffer deformation forces that destabilize prebiotic microsystems (Kompanichenko, 2017a, 2019). The relevance of those approaches is that molecular complexes can be incorporated into the assemblages to work like elements informing about external conditions. A consequence is that the resulting geometry of molecular entities is the basis for constructing interactions between emerging systems and surroundings. It is worth mentioning that such theoretical studies assume an unstable milieu for molecular structuring. Under far-from-equilibrium conditions, prebiotic systems are repeatedly disassembled or prone to acquire stable configurations among a miscellany of possibilities (Michaelian, 2017; Kompanichenko, 2017c). Unstable conditions exert critical influence over prebiotic systems so that there appear multiple stationary states supporting diverse behaviors toward environmental variables. Accordingly, the presence of an unstable milieu has been proposed as a necessary condition for the origin of life in addition to the presence of liquid water, organic material, and energy sources (Kompanichenko, 2019).

Many sorts of molecular complexes can support spatial information flow. However, under the harsh conditions that possibly dominated the prebiotic Earth, resilient peptide folding in nascent systems could confer resistance to milieu perturbations while interacting with it. Among the diversity of molecular structuring, the peptides could attain resilient conformations with the property to propagate these geometries (Maury, 2015; Cruz-Rosas et al., 2017). Because the resilient conformations are shaped in a particular time-lapse of the environmental oscillations, the pass of a system through a whole oscillation of unstable conditions could allow a reservoir of various resilient foldings. The reservoir can potentially work, buffering miscellaneous external perturbations if they have the property to induce the conformational change on malleable peptides into the system (Cruz-Rosas et al., 2017). The transfer of resilient conformations is the basis for a flow of spatial (conformational) information, which can be incorporated into the system architecture as dynamical scaffolding. In the beginning, this incipient adaptive behavior is driven by environmental oscillations. External stimuli induce the change of system states. At each lapse of milieu fluctuations, the retraction of pressure over some conformations enables its propagation while other conformations are destabilized, going into the set of malleable peptides. The above triggers system buffering of disturbances by collectively dissipating the absorbed energy. Hence, the basis for active system response and the construction of interactions with surroundings is settled.

### Information and Chirality

The spatial coupling between enduring peptides depends on geometrical restrictions. In this sense, the chirality of the amino acid constituents is a critical attribute in this prebiotic scenario. Even more, supported by the current homochirality, life optimizes its information flow by employing nucleic acids and peptides (Carroll, 2009; Cruz-Rosas et al., 2020). Because homochirality is a functional property and not only a biological signature (Korenić et al., 2020), both life and biochirality should have resulted from the same prebiotic event of symmetry breaking. It is important to note that several contributions have shown the plausibility of a chiral-asymmetric environment on prebiotic Earth (Brack, 2007; González-Campo and Amabilino, 2013; Pavlov and Klabunovskii, 2014; Burton and Berger, 2018). Furthermore, the observed extraterrestrial handedness on carbonaceous chondrites shows that chiral-asymmetric systems have a wide occurrence in the Universe and are not restricted to terrestrial conditions (Burton and Berger, 2018).

The presence of chiral-asymmetric locations on prebiotic Earth is a plausible assumption for our theoretical scenario. Accordingly, a functional flow of conformational information under unstable conditions emerges over the basis of the matching between peptides with similar inner enantiomeric purity (not necessarily homochiral sequences). Handedness makes a rate for spatial coupling possible between resilient conformations capable of counteracting the surroundings’ fluctuations (Cruz-Rosas et al., 2017). Therefore, in pre-life scenarios, the conformational information’s functional dynamic holds both system unity and chiral-asymmetry (chiral order). This potentially biogenic dynamic is the location where chirality and information establish an inherent linkage.

### Structuring Prebiotic Systems

Analogous to molecular structuring, prebiotic systems are also structured through environmental influence. The resulting system organization is a spatial one, promoting and restricting component interactions while molecular assemblage is developed. In this sense, the mere consideration of aggregation of peptide chains leaves out the environmental context. An essential aspect of our hypothesis is the role that surroundings exert over molecules and, in the view of the present work, drive system structuring because the environment also plays the role of a data resource by means of its represented parameters in the molecular shape. The polymers aggregation is only a starting point for our hypothesis. System organization supporting collective behaviors and interactions with surroundings is the focus of this current analysis.

**Figure 1:**
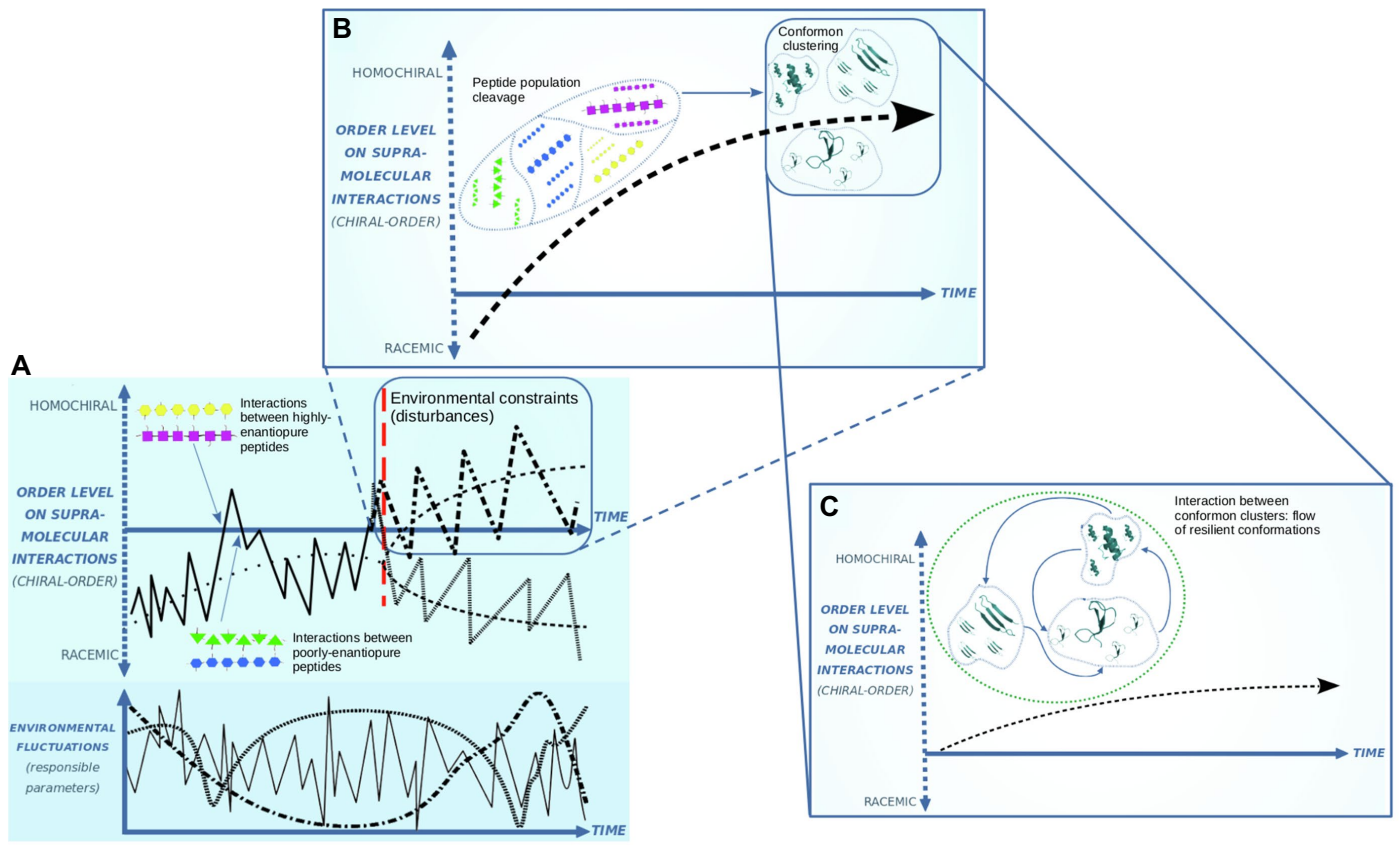
Clustering of enantiopure peptides and chiral-asymmetric systems formation. Under far from-equilibrium-conditions, chiral asymmetry is determining supramolecular interaction rates between peptides **(A)**. Environmental disturbances induce the cleavage on peptide populations, clustering the equivalently enantiopure conformons **(B)**. Structural destabilization and conformational change induction on the conformons allow an exchange of molecular shapes between clusters **(C)**.

Assembled under chiral-asymmetric conditions as a critical parameter of the unstable environment, prebiotic systems could incorporate peptides with a diverse degree of enantiomeric purity. For peptide prebiotic synthesis, the preferential incorporation of L-amino acids makes a variety of inner enantiopurities possible ranging between racemic and homochiral polymers (Figure 1A). Structured enduring peptides are opposing destabilization while at the same time represent the prevailing environmental conditions. In current living systems, spatial coupling and conformational change induction are biological information elements (Cruz-Rosas et al., 2020). The informational dynamic based on the adoption and propagation of a resilient conformation is observed on current prions. The contemporary prion dynamic is under the control of the cell conditions. Prion propagation efficiency runs over the existence of a specific sequence of amino acids by the genetic code. Homochirality in prions results from ribosome activity. However, this condition is not invalidating our hypothesis: We have addressed that homochirality optimizes the flow of conformational information. In this sense, the same protein sequence and handedness reinforce this optimal flow of conformational information in prion propagation. Accordingly, resilient and self-propagating prion-like conformations have been proposed to support a potential biogenic dynamic (Ogayar and Sánchez-Pérez, 1998; Maury, 2015, 2018; Cruz-Rosas et al., 2017, 2020).

The current conditions in living systems support the optimal flow of this explicit information based on spatial matching. In prebiotic scenarios, presumably without homochirality, ribosome activity or genetic code, the flow of conformational information was not optimal. Even more, if only chirality among the above conditions is playing at the origin of life, homochirality works as an unreachable upper limit for the progressive optimization of the flow of conformational information in prebiotic systems (Figure 1A). The prion propagation example reveals the current optimization conditions for conformational information functionality and supports the affirmation that (homo) chirality is related to improving the conformational information flow. The general assumption in the presented work here is that the first (functional) prebiotic information was the explicit conformational information (Cruz-Rosas et al., 2017, 2020). The argument of the prebiotic emergence of *functional* information is based on a system response toward the milieu. In this sense, the propagation of resilient conformations must stabilize the system scaffolding and allow an incipient adaptive behavior, buffering the physicochemical disturbances.

At this point, it is relevant to clarify that our hypothesis is about the conformational information, which becomes functional over the basis of chiral asymmetry when prebiotic systems are assembled under sharp environmental variations. This theoretical scenario represents a bottleneck for the emergence of potentially biogenic dynamics: Those with adaptive behavior supported by the transfer of conformational information. We took the particular way of peptides, which are informational molecules in the context of the current hypothesis. Of course, a scenario based on nucleic acids is also possible in our approach. In that case, RNA was an entity carrying geometrical information in the early informational dynamics. We have put in context the capability of RNA to carry this spatial information (Cruz-Rosas et al., 2020). The need for inner enantiomeric purity was also a critical condition. Thus, if chiral symmetry breaking was first on RNA, it was a functional property (Korenić et al., 2020) then transferred to peptides. The sequential-genetic information in contemporary living systems gives a clue to consider that nucleic acids are better to carry the sequential information than the spatial one. Therefore, we start from peptides supporting confrontational information dynamics as a previous step for the emergence of codifying sequences of nucleic acids.

Our theoretical scenario is not addressing the origin of enantiomeric excess over raw material such as in Viedma or Noorduin models (Viedma, 2007; Noorduin et al., 2008). Even more, we are not proposing a mechanism for amplification from a slight handedness, such as in the Soai reaction (Soai and Kawasaki, 2008). The theoretical scenario that we propose assumes the viability of these mechanisms, which have been validated utilizing models and experimental data. The availability of chiral-asymmetric locations on prebiotic Earth is an assumption in our approach. Hence, we are theoretically working over the consequences of interactions between polymers already owning nonracemic proportion on their constituent monomers (inner enantiomeric purity).

Incessant assemblage and disassembly of prebiotic systems allows a variety of configurations and (if any) their associated behaviors. Exerted dynamism from the unstable milieu is a condition to structuring prebiotic systems and a necessary condition to the origins of life (Michaelian, 2017; Kompanichenko, 2017a). In this context, a dynamic of conformational information flow must show a rate capable of counteracting the changing milieu’s disturbances. The peptide formation reactions (e.g., Dubina et al., 2013; Raos and Bermanec, 2017) could incorporate unsymmetrical enantiomeric proportions of chiral amino acids from the prebiotic environment. The resulting peptides have different (inner) enantiomeric purity, ranging between (all) racemic and (all) homochiral as (unreachable) limits (Cruz-Rosas et al., 2017). When incorporated into a dynamic of transfer of conformational information, inner enantiopurity determines the spatial coupling velocity between peptides.

The supramolecular matching between poorly enantiopure peptides is slower than peptides with high enantiopurity (Cruz-Rosas et al., 2017). Accordingly, during the construction of interactions by prebiotic systems toward unstable milieu, poorly enantiopure peptides owning resilient copying-conformations cannot transfer their spatial references at a rate able to counteract the external variations. Therefore, high enantiopure peptides can support a rate of conformational information flow to respond toward perturbations from the milieu oscillations. Under unstable conditions, chiral asymmetry makes the dynamic of conformational information functional.

Figure 1 depicts three stages for the emergence of adaptive systems based on conformational information and chiral asymmetry. Nonracemic molecular proportions are part of the far-from-equilibrium conditions in prebiotic Earth. Unbalanced oscillations of parameters such as temperature, salinity, pH, and pK (Kompanichenko, 2017b) destabilize the self-assembled molecular systems. Exerting pressure on a pool of peptides, environmental fluctuations destabilize conformations that do not establish successful couplings to acquire resilient conformations, exposing some peptides to unfolding or breakdown. New peptide structuring at another point of the milieu variations allows testing new folding to acquire resilient and spreadable conformations. These hypothetical prebiotic peptides that acquire the capability to self-propagate their conformations are called *conformons* (Ogayar and Sánchez-Pérez, 1998). Sharp variations in milieu conditions make a rate for propagating these resilient conformations necessary to counteract disturbances collectively. This rate runs on spatial matching, which depends on geometrical restrictions. As stated, the main geometrical restriction to coupling polymers is the handedness in its constituent monomers.

Peptides with inner enantiomeric purity (chiral order) are prone to couple with equivalent enantiopure peptides. Fluctuating environmental constraints can surpass or not the rate of peptide supramolecular matching. Therefore, a rate for matching based on high enantiopure peptides will propagate the resilient conformations between peptides to buffer the environmental disturbances. The above is conducted in Figure 1A, which shows a bifurcation event where peptide populations define two branches: The upper one keeps and increases the chiral order in these polymers because they are propagating resilient conformations as enantiomeric purity optimizes their supramolecular coupling (making the flow of conformational information functional). Moreover, the decreasing branch shows the loss of chiral order due to the matchings run on geometric difficulties. The decreasing branch is conducted toward the racemic condition, where conformational information is unable to be functional. Fluctuating environmental conditions, working as constraints for peptide maintenance, overpass the rate for transferring resilient conformation among these near racemic polymers, so they are disassembled.

Figure 1B considers only the upper branch. It is depicting a stage of clustering of peptides over the base of their inner enantiopurity. This peptide clustering brings folded chains closer because spatial coupling shows a rate capable of counteracting milieu disturbances employing resilient conformons (Cruz-Rosas et al., 2017). Throughout the environmental oscillations, the peptide clustering in a pool will define diverse clusters over the base of each type of resilient conformon. Although they are enduring peptides, milieu perturbations have effects over conformon clumps so that there is a peptide-disassemble rate for each cluster. The disassemble rate makes available a resource of peptides for refolding and its incorporation into some clusters. This incorporation of new refolded peptides depends on the successful transfer of the spatial references, so chiral asymmetry is maintained. Increasing enantiomeric excess in a cluster is attained by incorporating peptides with significant inner enantiomeric excess that increases the rate of supramolecular coupling.

**Figure 2:**
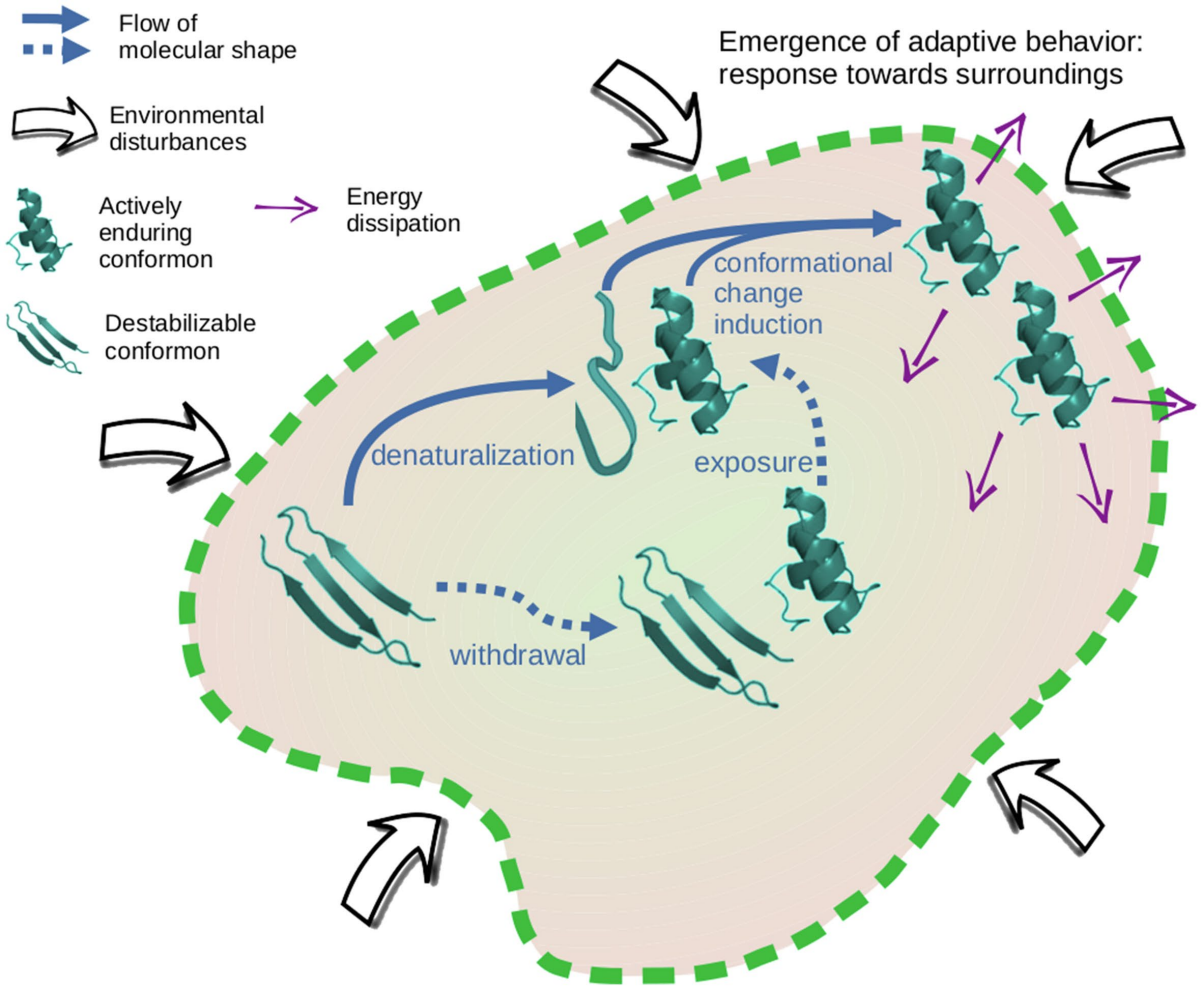
Structuring adaptive prebiotic systems through the conformational information. The flow of conformational information between clusters permits the structuring of a dynamical scaffolding that supports system interactions with surroundings in an emergent adaptive behavior.

Over a time span, the formation of a conformon clump (maybe with only a few members) works as a reservoir of resilient and spreadable conformations to support a diversity of interactions with surroundings. When milieu conditions stress on a cluster, its population number begins to decrease while another conformon cluster can maintain its conformation and propagate it both to denatured peptides and destabilized conformons. This assumes the closeness between clusters, enabling an exchange of peptides and, most of all, a flow of conformational information transiting toward a system level of organization (Figure 1C). Consequently, the resiliency of a conformon cluster at a point of the environmental oscillation may help buffer the effects that upset the stability of another cluster that has entered into a destabilization dynamic. The above could be supported by a mechanism for absorbing the energy contained in the environmental parameters by the enduring conformons to dissipate it toward the surroundings. Accordingly, the conformon cluster’s members who actively resist and propagate stay directly exposed to the surroundings while the destabilized cluster’s members remain indirectly exposed (Figure 2). This dynamic based on enduring conformon clusters could be contained in a system with a physical barrier such as a membrane or inside a mineral substrate’s pore. The prebiotic microsystems structured in this way show a flow of conformational information at a system level of organization (bringing a variety of enantiopure conformon clumps together).

In this scenario, system structuring can be identified where the clusters of enduring peptides are buffering the milieu disturbances at several points of the environmental oscillations. The direct exposure of actively resilient conformons toward the environmental parameters allows the microsystem to start constructing a response toward surroundings (Figure 2). This dynamical scaffolding is supporting an adaptive behavior of the microsystem as an integrated whole.

### Inheritance Supported by Self-Propagating Conformations

Genetic polymers support biological inheritance. Crucial processes such as replicating nucleic acids need high accuracy to ensure the transference of the sequential information from a cell to progeny. On an evolutionary timescale, replicating some sequential-information unities to reach a high frequency in a biological population is the basis for Darwinian evolution. The increasing complexity in this trend has been proposed to be a consequence of storing (sequential) information in nucleic acids (Szostak, 2017). The emergence of that Darwinian evolutionary way has been addressed as the fundamental step in the origin of life (Szostak, 2017; Kunnev, 2020). In pre-life dynamics, replication and spreading of random sequences in a population of polymers is not necessarily an evolutionary trend because not all sequences are informational entities. Focusing on individual randomly generated sequences would lose sight of the property of carrying information in prebiotic scenarios. In our theoretical approach, replicating a particular conformation and its resulting high frequency in a peptide population is not the objective. Conversely, due to spatial structuring containing information about particular surrounding conditions, the diversity of conformations is postulated as imperative in our hypothesis. The above allows systems to acquire diverse data referring to the milieu variations on a time-lapse. The propagation of resilient conformations is not to dominate within the system but to support a flow of conformational information to interact with the changing surroundings.

Self-replication has been shown on amyloid structures (Maury, 2018). Even so, this capability is only on the ground for the emergence of an informational system. Peptides carry conformational information, so the sequential information is not a necessary feature in these polymers but a property acquired by oligonucleotides that could be incorporated into a system with a flow of spatial information. The arising of coding sequences is a postulated consequence of the stability inside of this class of adaptive systems. We theoretically discuss the occurrence of a particular prebiotic inheritance centered on enduring conformons with the ability to self-organize scaffolding capable of exposing resilient conformations to propose interactions with surroundings in the preceding described manner.

Clusters of conformons construct links between them by the exchange of conformations. This swap is motivated by milieu constraints that destabilize some peptides to be prone to refolding. The cluster connection determines a unity capable of interacting with surroundings. Once this dynamical scaffolding is reached, the flow of conformational information simultaneously occurs supported by it. The mere propagation of a specific conformation is insufficient to support an informational dynamic because it cannot contribute to the collective response. In this sense, inheritance in our hypothesis is possible only if a set of diverse conformations is given down toward derivative systems so that this set of enduring peptides can build and organize a network of interactions. The propagation of resilient foldings to system periphery in the informational dynamic is the groundwork for buffering the milieu disturbances (Figure 2). This adaptive behavior becomes the entity subject to explore evolutionary transitions toward the origin of living systems.

Although the system structuring allows the buffering of external perturbations, the microsystem is not exempt from physical breaking. In an unstable milieu, the physical barrier or the internal cohesion may be disrupted. The breaking of prebiotic microsystems has been proposed as a simple mechanism to propagate the achieved structuring or dynamic in a molecular consortium (Herrera, 1942; Cornish-Bowden and Cárdenas, 2008). Therefore, the structuring in which actively resilient conformations move toward the periphery to support interactions with the surroundings (Figure 2) could be inherited (not yet in a biological sense) through microsystem breakage. The subsequent arising of functional sequences (in genetic polymers) within this class of systems was an advantageous property to trigger the (genetic) development of life. Consequently, the potential to structuring systems over the base of a flow of conformational information was a bottleneck for the emergence of biogenic dynamics.

Each microsystem derived from the breakage of a preceding one with dynamical scaffolding begins its autonomous existence with a reservoir of diverse types of enduring conformons in such a way that it is possible to structure them in the terms mentioned above to favor the emergence of adaptive behavior (Figure 2). The inheritance of the set of conformons that support the transfer of molecular shapes as informational data allows the inheritance of the potential capacity for the self-structuring of the system. Consequently, based on conformational information, it is necessary to inherit a reservoir of diverse conformons by system breakage to make the subsequent microsystem structuring for adaptive behavior possible.

### Early Steps to the Development of Functional Sequences

The emergence of functional sequences in overall environmental conditions seems unlikely because their meaning is strongly order-dependent. This order on sequences has the trend to be decreased in favor of the increase of the uncertainty. Unless there exists a context or process determining the ordering, milieu conditions disrupt or erase any meaning of the sequences. To avoid this problem, the hypothesis of spatial information is an alternative that brings a new location to consider the origin of genetic information within a restricted environment and a reduced pool of amino acids. In this sense, several hypotheses state that the origin of coding sequences occurred in the recurrent interactions between amino acids and random RNA sequences. These interactions may have been guided by functional properties, such as optimization of the absorption of UV light by nucleotides, in the context of the dissipation of the irradiated energy from the Sun (Michaelian, 2017). Besides, the origin of coding sequences may have been based on chemical affinities and structural stabilization to oligonucleotides associated with amino acids (Banfalvi, 2019).

Based on our hypothesis, the interactions between amino acids and random sequences of RNA took place in the systems capable of interacting with the milieu in the here presented way. To attain the spatial coupling with L-amino acids, the functional dynamic of conformational information (based on chiral asymmetry) also provides the chiral bias for the initial favoring of RNA based on D-ribose. It is important to have in mind that in our approach, chiral asymmetry is a necessary condition. In this sense, the dominant handedness in a microsystem with functional conformational information flow was crucial for developing sequential (genetic) information because the chiral asymmetry is supporting an optimal flow of this cryptic information in codifying sequences (Carroll, 2009). The context of spatial information for the development of coding sequences represents an initial response to the research question about how peptides and oligonucleotides were associated to configure a biogenic system (Kunnev, 2020). Consequently, the possible (proto-) genetic interactions concerning amino acids and RNA sequences were restricted to locations in microsystems owning conformational information supported by the chiral bias.

The data from ordered sequences trigger processes to interact with (respond to) internal or external system context. Besides the flow of sequential information between nucleic acids, a central aspect of this cryptic biological information is protein synthesis. A transducer (the ribosome) links sequential data in nucleic acids with the resulting peptide chain. The arising of this cryptic information assumes a basic set of rules to decode the meaning in a polymer. In this sense, the origin of the ribosome should be posterior to the appearance of nucleic acid sequences. The ribosome is a linking component defining the correspondence between sequences of nucleotides and amino acids. Conclusions stating that the ribosome essentially is an RNA machine and also that the evolution of the ribosomal decoding region (the peptidyl transferase center) was not driven by its protein components but due to the enhancement of the rRNA (Fox, 2010) are consistent with our scenario where nucleic acids carry sequential information and peptides carry the spatial one. In the same sense, the affirmation that the structural motifs were incorporated from already folded peptides to constitute its small and large subunits (Fox, 2010) supports the idea that the origin of ribosome and, consequently, the development of meaning in the sequences, may have occurred in stabilized locations at the interior of the here described adaptive systems.

It is important to notice that we are not proposing a linear process from conformational to sequential information but diverse prebiotic systems containing some spatial information flow dynamics. In this sense, the heterogeneity of prebiotic assemblages could be the basis for the interaction between conformons and potentially genetic polymers. The incorporation of oligonucleotides and their associations with (peptidic) conformons allowed the construction of new interactions with surroundings by these potentially biogenic systems. Thereby, oligonucleotides with random sequences could be structurally stabilized and standardized toward the emergence of coding sequences and the rules to interpret them (decipher them) in the system context. Probing several variants of interactions with surroundings, this type of putative biogenic systems could be the starting point toward the evolution of primitive life, initiating the genetic development to the biological lineages.

## Remarks

Adaptive behavior in prebiotic microsystems may emerge from a system structuring supported by the conformational information dynamic. In this context, enduring peptides working as conformons can propagate their resilient conformations. This informational dynamic allows microsystems to buffer external disturbances by dissipating absorbed energy from environmental parameters. Consequently, these prebiotic systems can construct interactions with their surroundings.

In an unstable environment with sharp oscillations, the conformational information based on peptides owning enantiomeric bias in its constituent amino acids becomes functional. This inner-handedness lets resilient conformations propagate at a rate that permits the system to counteract the milieu influences. Chiral asymmetry contributed to the optimum flow of information, both conformational and sequential (Carroll, 2009; Cruz-Rosas et al., 2017).

The diversity of resilient conformations reflects the different points in the milieu oscillations, which works as a reservoir of enduring conformons for the prebiotic microsystems that contain them and support the construction of relationships between the system and surroundings at several times.

A dynamical scaffolding in a system may be induced by a reservoir of enduring (and spreadable) conformons. This dynamical architecture’s main feature is the direct exposure of actively resilient conformations to the milieu conditions; simultaneously, the destabilized conformations are withdrawn to internal system locations. When the environment changes, there is a rearrangement of resilient conformons that trigger an active response toward the milieu. This system structuring was the cradle for the stabilization of sequences of RNA.

Interactions between RNA oligonucleotides and amino acids of conformons could have reinforced or initiated new functional properties in the system, such as improving energy dissipation from environmental disturbances. The origin of a transducer (the ribosome) may have taken place in this context.

The current theoretical approach expands our general framework to the emergence of adaptive systems. Accordingly, our proposal addresses this necessary condition for the arising of coding sequences. Hence, over the base of our approach, it is possible to design *in silico* or mathematical models to search for evidence to validate our theory at a system overview.

## Data Availability Statement

The original contributions presented in the study are included in the article/supplementary material, further inquiries can be directed to the corresponding authors.

## Author Contributions

HC-R and PM contributed to conception and design of the study, wrote the first draft of the manuscript, and contributed to manuscript revision, read, and approved the submitted version. All authors contributed to the article and approved the submitted version.

## Funding

The DGAPA-UNAM postdoctoral fellowship financially supported this work to HC-R.

## Conflict of Interest

The authors declare that the research was conducted in the absence of any commercial or financial relationships that could be construed as a potential conflict of interest.

## Publisher’s Note

All claims expressed in this article are solely those of the authors and do not necessarily represent those of their affiliated organizations, or those of the publisher, the editors and the reviewers. Any product that may be evaluated in this article, or claim that may be made by its manufacturer, is not guaranteed or endorsed by the publisher.
